# Droxidopa as an effective treatment for refractory neurogenic orthostatic hypotension and reflex bradycardia in amyloid light-chain amyloidosis: a case report

**DOI:** 10.1186/s13256-020-02405-w

**Published:** 2020-06-20

**Authors:** Annie H. Ho, Christopher W. Kinter, John Wight, Anudeep R. Neelam, David Krakow

**Affiliations:** Department of Medicine, Emory University School of Medicine, Atlanta, GA USA

**Keywords:** Droxidopa, Neurogenic orthostatic hypotension, Amyloidosis, Case report, Reflex bradycardia

## Abstract

**Background:**

Droxidopa is an oral treatment for the stepwise treatment of neurogenic orthostatic hypotension from autonomic dysfunction. It has been shown to be useful predominantly with neurogenic orthostatic hypotension secondary to Parkinson’s disease, but only a few cases have documented its usefulness in patients with neurogenic orthostatic hypotension due to amyloidosis, which is often severe and refractory. In addition, only one source in the literature reports the concomitant use of midodrine and droxidopa for such patients. Finally, we argue that droxidopa seems to have a protective effect against episodes of reflex bradycardia, which is not previously reported.

**Case presentation:**

A 64-year-old white man was admitted for 1 year of worsening syncopal episodes, diarrhea, failure to thrive, heart failure, and neuropathy. Medical emergencies were called five times on the overhead hospital intercom over a 4-day period in the beginning of his admission due to severe hypotension and bradycardia. He was eventually diagnosed as having amyloid light-chain amyloidosis and myeloma. After starting droxidopa, both his systolic blood pressure and reflex bradycardia improved, and no more medical emergency events were called during the remaining 30 days of admission. He felt much better subjectively and was able to sit upright and engage in physical therapy.

**Conclusions:**

We show that droxidopa is effective when used with midodrine to treat refractory neurogenic orthostatic hypotension in patients with amyloidosis. There are very few cases reporting the use of droxidopa in amyloidosis, with only one study that uses droxidopa and midodrine concomitantly. In addition, our patient’s reflex bradycardia improved drastically after starting droxidopa, which we believe is mediated by increased systemic norepinephrine. There were no side effects to droxidopa, and the benefits lasted well beyond the reported duration of 1–2 weeks that was noted to be a limitation in some studies.

## Background

Amyloid light-chain (AL) amyloidosis is a disease that occurs in 9 cases per million person-years, with roughly 2200 new cases of AL amyloidosis annually in the USA [1]. The disease is characterized by plasma cell production of aberrantly folded light chains. The light chains deposit around the body, causing organ dysfunction. Common features are kidney dysfunction, heart failure, arrhythmias, gastrointestinal issues, fatigue, hepatomegaly, spleen dysfunction, peripheral neuropathy, and orthostatic hypotension [2]. Orthostatic hypotension can be a particularly debilitating aspect of the disease that is difficult to manage [2]. The mainstay of treatment options have been midodrine and fludrocortisone, however, fludrocortisone is often not an option in the case of heart failure due to the fluid retention it causes.

In 2014, the US Food & Drug Administration (FDA) approved a novel oral therapeutic called droxidopa for the treatment of neurogenic orthostatic hypotension (nOH). nOH is a type of orthostatic hypotension that is characterized by severe orthostatic hypotension, compromised recovery to normal blood pressures (BPs) following vagal maneuvers, and diminished heart rate (HR) response to hypotension [3]. Droxidopa is expensive without insurance coverage, costing up to nearly US$8000 for a 30-day supply of the highest dose according to GoodRx (a US company that tracks prescription drug prices). However, it is very affordable with many insurance plans, and was around US$40 a month for our patient in this case. It is a synthetic peptide that is converted peripherally and centrally to norepinephrine [4, 5]. A proposed mechanism for nOH is that there may be a systemic deficit of norepinephrine [6], thus making droxidopa a sensible treatment.

In a meta-analysis, droxidopa resulted in increased upright systolic blood pressure (SBP) by 11.5 ± 20.5 mmHg in addition to improved activities of daily living (ADL), orthostatic symptoms, visual disturbances, weakness, and fatigue [7]. There were 68% fewer falls recorded in those treated with droxidopa versus placebo. However, the studies only noted significant improvement over 1-week to 2-week periods, whereas longer usage failed to show significant benefit [8].

Droxidopa has been used for various causes of orthostatic hypotension such as Parkinson’s disease, pure autonomic failure, multiple system atrophy, nondiabetic autonomic neuropathy, and dopamine β-hydroxylase deficiency [9]. One study found that patients with nOH due to amyloidosis had significantly more severe symptoms than those with nOH due to other etiologies and, therefore, required higher doses of droxidopa [10]. This is likely because patients with amyloidosis can have hypotension due to issues beyond nOH. They also often have coexisting amyloid cardiomyopathy, volume depletion from diarrhea, and drug side effects (such as those to treat heart failure) [11]. In the study of droxidopa in critically ill patients with amyloidosis, four of six patients were refractory to midodrine, but the majority of these were successfully treated with droxidopa (five remained on treatment at 6 months) [10].

This case report is unusual for the severe manifestations of nOH in the setting of amyloidosis and for the striking improvement with the addition of droxidopa. We offer several new treatment strategies for severe, refractory nOH to contribute to the medical literature. Novel aspects of this report are the following: droxidopa may be synergistic with midodrine, the beneficial effects of droxidopa can last longer than the 1–2 weeks previously reported, and droxidopa may have a protective effect against episodes of vagally mediated bradycardia.

## Case presentation

A 64-year-old white man presented to our emergency department (ED) for persistent weakness and autonomic dysfunction the day after a 27-day stay at an outside hospital where he was treated in the intensive care unit for septic shock due to *Escherichia coli* urinary tract infection. He was admitted to our internal medicine teaching service for further workup of his worsening dysautonomia.

His past medical history included 3 years of progressive failure to thrive, dysautonomia characterized by frequent syncopal episodes and neurogenic bladder, occasional volume overload, and chronic non-bloody diarrhea. The syncopal episodes began occurring 1 year prior to admission and were in the setting of bowel movements or standing. He was bed-bound at the time of admission and was pre-syncopal with sitting upright. He was taking 0.2 mg fludrocortisone twice a day (we held this on admission for heart failure symptoms) and 15 mg midodrine three times a day for syncope. He was noted to have pleural effusions and ascites at the outside hospital, and he was discharged with 40 mg orally administered furosemide daily. He was not on any beta-blocker. The diarrhea was roughly three times a day, watery, and yellow to brown in color. He was taking loperamide and pancrelipase for the diarrhea with minimal improvement.

His family history was relevant for heart disease starting at old age in his sister, brother, and father. His social history included a 20 pack-year smoking history but was negative for alcohol or illicit drug use. Before his previous hospitalization, he lived at home with his wife in a rural town, and he was able to complete his ADL with minimal assistance.

On arrival to our emergency department (ED), his vital signs were: 37 °C, blood pressure (BP) 62/42 mmHg, heart rate (HR) 75, respiratory rate 10 breaths per minute, and oxygen saturation of 85% on room air. Orthostatic BPs were not able to be obtained due to his weakness and lightheadedness with sitting upright even. His body mass index (BMI) was 21.6. On physical examination he was generally frail appearing, but in no distress. An examination of his head revealed temporal wasting and macroglossia. His heart had a regular rate and rhythm without murmurs or extra heart sounds, and he did not have jugular venous distention (JVD) or peripheral edema. Crackles were auscultated up to the mid-lung fields on the right and at the base on the left with moderate volume ascites. His abdomen was slightly distended with mild lateral bulging. Bowel sounds were present, and there was no rigidity or tenderness of his abdomen. On his neurologic examination, he was alert and oriented, without cranial nerve deficits, his strength was 4/5 in all extremities, deep tendon reflexes were slightly hypoactive in the patellar and Achilles tendons, sensation was decreased in all modalities in length-dependent distribution, and coordination was intact without dysmetria. He did not have clonus or an upgoing plantar reflex. We were not able to test gait due to debilitating weakness and orthostatic hypotension.

**Fig. 1:**
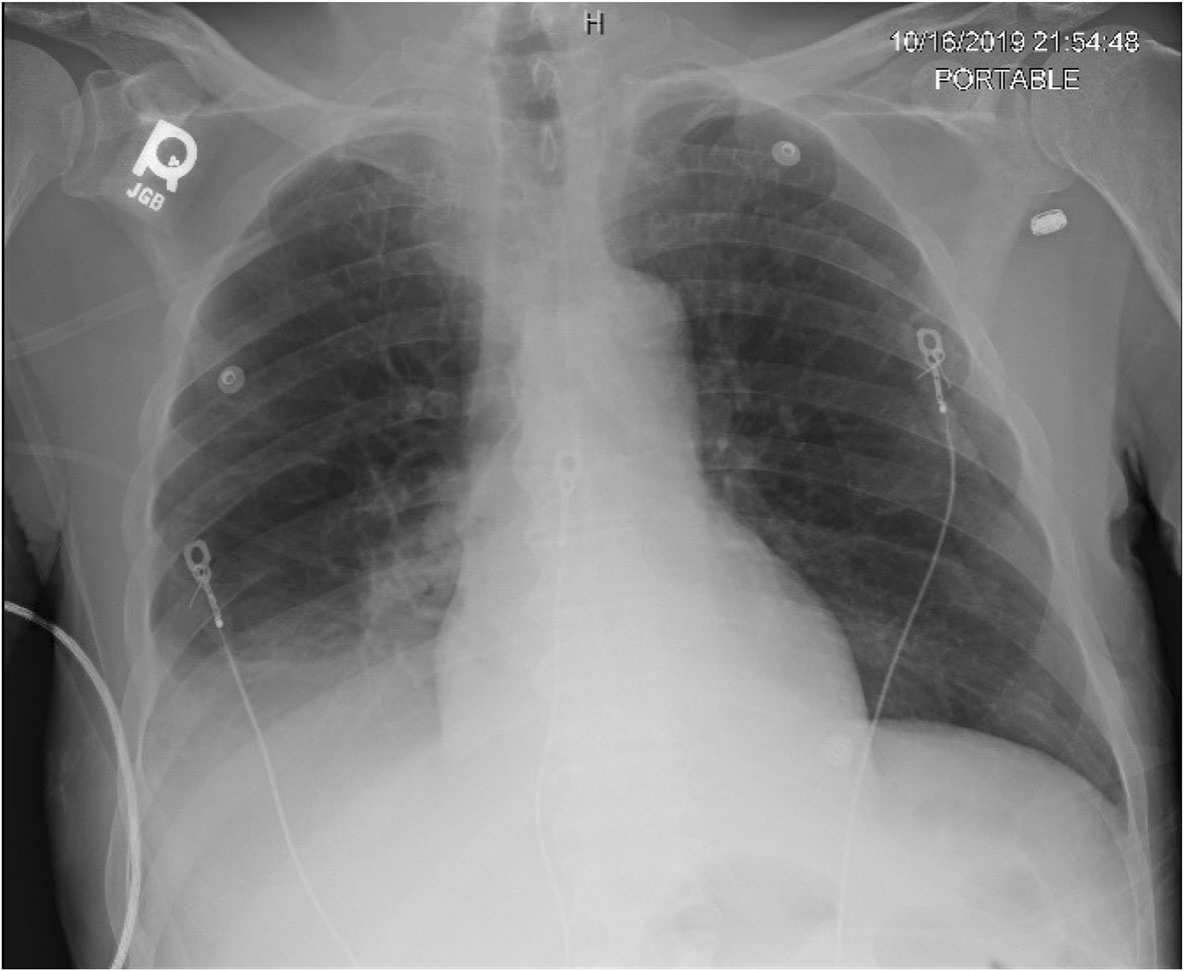
Anteroposterior chest X-ray showing a moderate right pleural effusion

**Fig. 2:**
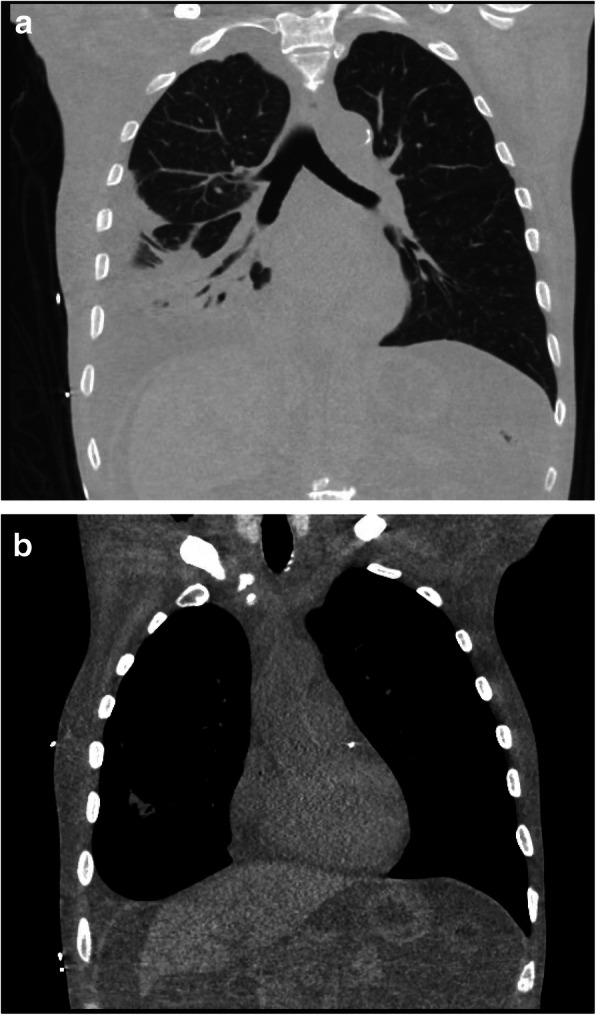
**a** and **b** Chest computed tomography with contrast showing large right pleural effusion (**a**) and large ascites (**b**)

Initial laboratory results were significant for white blood cell (WBC) count of 13.1 × 10^9^ cells/L with neutrophil predominance, 25 WBC and slight leukocyte esterase with 100 mg/dL protein on urine analysis (UA), glomerular filtration rate was calculated to be 51 mL/minute/1.73m^2^, calcium was normal, and a brain natriuretic peptide (BNP) of 1037 pg/mL. Plasma cortisol on admission was 30.1 mcg/dL (repeat on day 2 10.5) and adrenocorticotropic hormone (ACTH) levels (13.4 pg/mL) were within normal limits. A chest X-ray on admission showed a moderate right pleural effusion (Fig. 1). A computed tomography (CT) scan of his chest revealed large right and small left pleural effusions (Fig. 2a) and large abdominal ascites (Fig. 2b).

An electrocardiogram (EKG) was low voltage with left axis deviation and a right bundle branch block. An echocardiogram revealed severe eccentric left ventricular hypertrophy, mild dilation of the left atrium, with reduced ejection fraction of 35–40%, and a bright, speckled appearance of myocardium. Serum free lambda light chain was elevated at 1256.4 mg/L with a kappa to lambda ratio of 0.02. Initially, a fat pad biopsy was nondiagnostic. A subsequent bone marrow biopsy on hospital day 12 showed 10% plasma cells with lambda monotypic population on flow cytometry, marked extracellular eosinophilic deposits, and a focus of congophilic deposit, suggestive of amyloidosis and myeloma. Hematology came to the final diagnosis of AL amyloidosis and myeloma with nerve, cardiac, and gastrointestinal involvement.

The initial hypotension corrected to 128/87 mmHg 12 minutes after the initial reading without intervention. His pulse remained stable at 87 beats per minute. He was then given 1 L of intravenous Lactated Ringer’s solution and 10 mg intravenously administered dexamethasone and his BP remained stable until hospital day 2 when he had a BP of 84/44 mmHg with a pulse of 71 beats per minute that was not announced to the primary team as a medical emergency.

The main goal for the first few days of admission, before we knew he had amyloidosis, was to treat his heart failure and malnutrition. Because he was clinically volume overloaded with pleural effusions, hypoxia, and an elevated BNP, we gave 40 mg orally administered furosemide on the first 3 days followed by 20 mg torsemide for the next 2 days. We were balancing fluid loss from diuretics and diarrhea with 2 L of peripheral parenteral nutrition (PPN) per day because he had malnutrition estimated as severe based on the Academy of Nutrition and Dietetics (Academy)/American Society for Parenteral and Enteral Nutrition (ASPEN) clinical characteristics. He was net negative approximately 1 liter per day for the first 4 days of his hospitalization. One dose of cefepime was given in our ED, but our team did not elect to continue antibiotics as his UA was not concerning and his blood and urine cultures remained negative while his WBC count normalized.

On hospital day 2, he had two episodes of hypotension (84/44 mmHg and 71/51 mmHg later in the day) with normal pulses (71 beats per minute and 63 beats per minute, respectively). No alert for a medical emergency was called for either of these readings, and the resident on call responded by giving 1 L of intravenously administered fluids. We later increased his midodrine dose to 20 mg three times a day and his BP improved. He was not tachycardic and his creatinine was improving with diuresis (1.26 on admission to 1.04). He did not display contraction alkalosis to suggest that he was volume depleted. His WBC count had normalized by day 4 without antibiotics since the ED dose of cefepime.

Two days went by where he was stable and no changes were made, but on day 5 he started to have frequent episodes of severely symptomatic nOH with unmeasurably low BP using the electronic cuff (as low as 44/25 mmHg on manual checks) and bradycardia in the range 40s to 50s beats per minute. Each episode was associated with a bowel movement or taking medications, leading to five medical emergency events being called on the hospital overhead speaker over a 4-day period.

We considered the diuretics and diarrhea as potential exacerbators of these hypotensive episodes, so we stopped torsemide and increased his 4 mg loperamide from daily to three times a day. He was roughly net negative approximately 4 liters at the time the hypotensive and bradycardic episodes began occurring. However, without tachycardia, contraction alkalosis, or acute kidney injury we felt it unlikely that these episodes were due to volume depletion. After the fifth call for medical emergency for hypotension, on hospital day 8, we started 200 mg droxidopa three times a day.

We felt that this was not volume depletion because our patient was still volume overloaded at this point. Serum creatinine had improved with gentle diuresis, and there was no contraction alkalosis noted on laboratory results. Also, our patient was not persistently hypotensive, as one would expect if volume depleted. Further, he was bradycardic during episodes, and the episodes were clearly exacerbated by vagal maneuvers (bowel movements and one time when taking medications). He often took 10 to 20 minutes to recover to a normal HR from his bradycardia, and the drops in his BP were severe and sudden. We had previously ruled out adrenal insufficiency with normal ACTH and cortisol levels, and the events seemed too episodic to be consistent with the diagnosis. Therefore, we concluded that this was nOH secondary to amyloidosis.

**Fig. 3:**
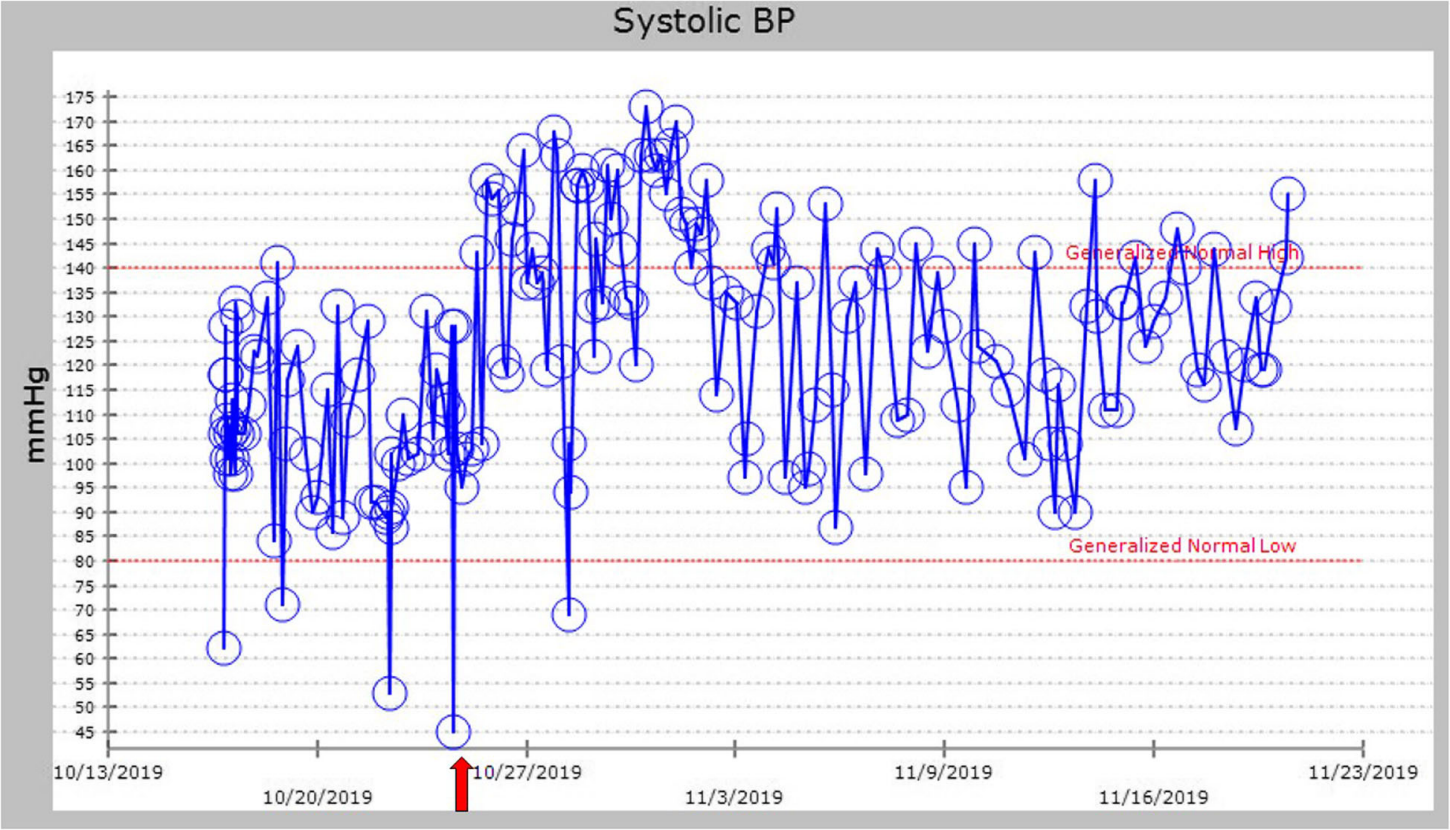
Trend showing improvement of systolic blood pressure readings during admission. The *red arrow* denotes droxidopa start date. *BP* blood pressure

**Fig. 4:**
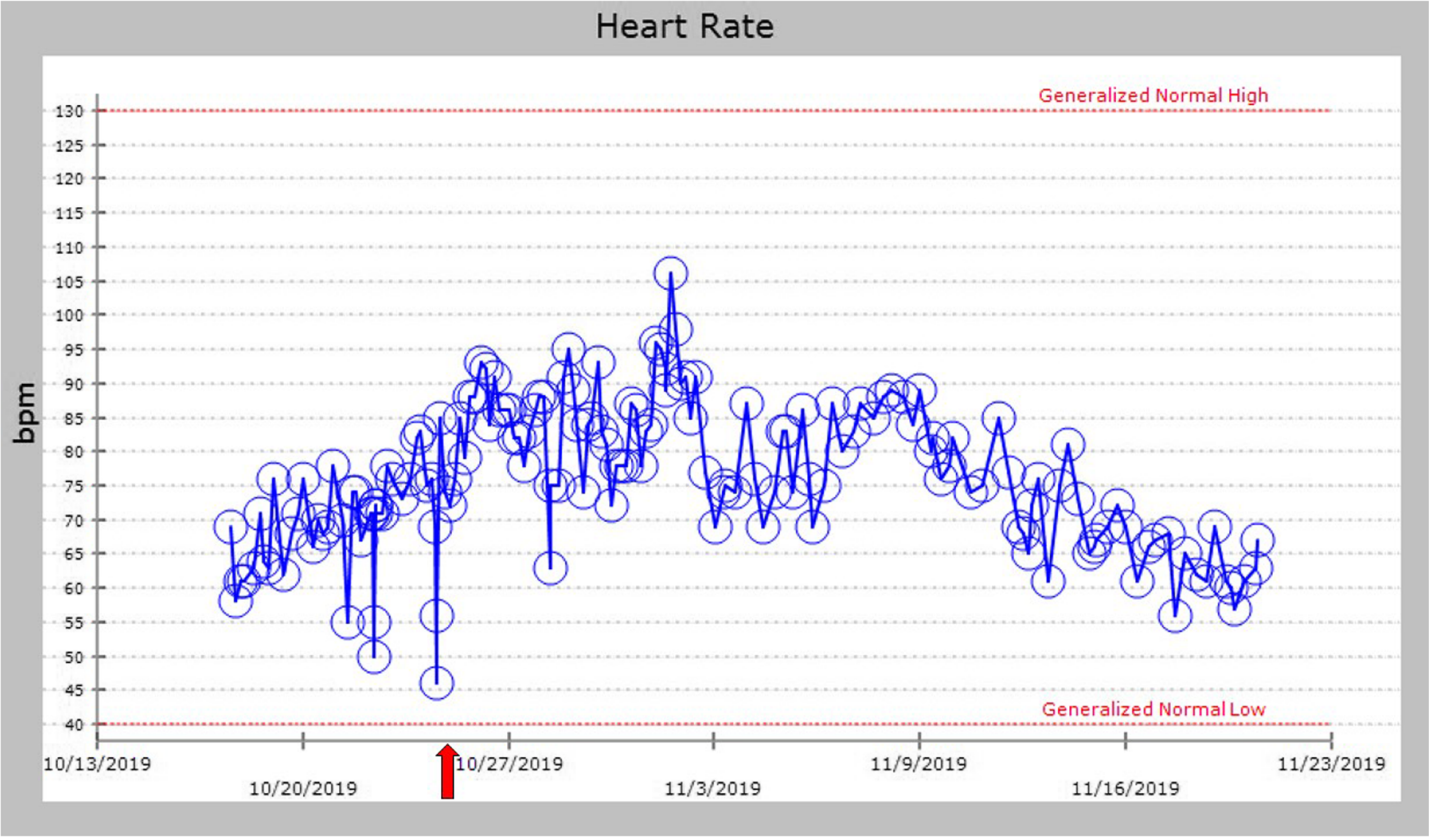
Trend showing improvement in episodes of bradycardia during admission. The *red arrow* denotes droxidopa start date. *bpm* beats per minute

His BP remained improved during the first day after starting droxidopa, and his dose was increased to 300 mg three times a day because some of his SBP readings were still in the low 90s. Droxidopa was the only medication that was changed during this time, and all other medications and fluids were continued at the same doses and rates. By the third day of treatment, he felt much more energetic and was able to sit upright in a chair, which he had been unable to do for over a month. A plot of his SBPs shows how he improved after starting droxidopa (Fig. 3) (Unfortunately, several of the unmeasurably low BP readings from the automated BP cuff were not recorded in the electronic medical record, thus are not reflected in the plot). Also, his HR improved, without subsequent bradycardic episodes (Fig. 4).

He was transferred to the oncology service on hospital day 14, and he was started on bortezomib, cyclophosphamide, and dexamethasone on day 19. He was noted to develop an elevated WBC count at the time of his transfer, and on day 19 he tested positive for *Clostridium difficile.* Treatment with orally administered vancomycin was completed, however, he still had significant diarrhea after 10 days. However, his pressures remained stable.

**Fig. 5:**
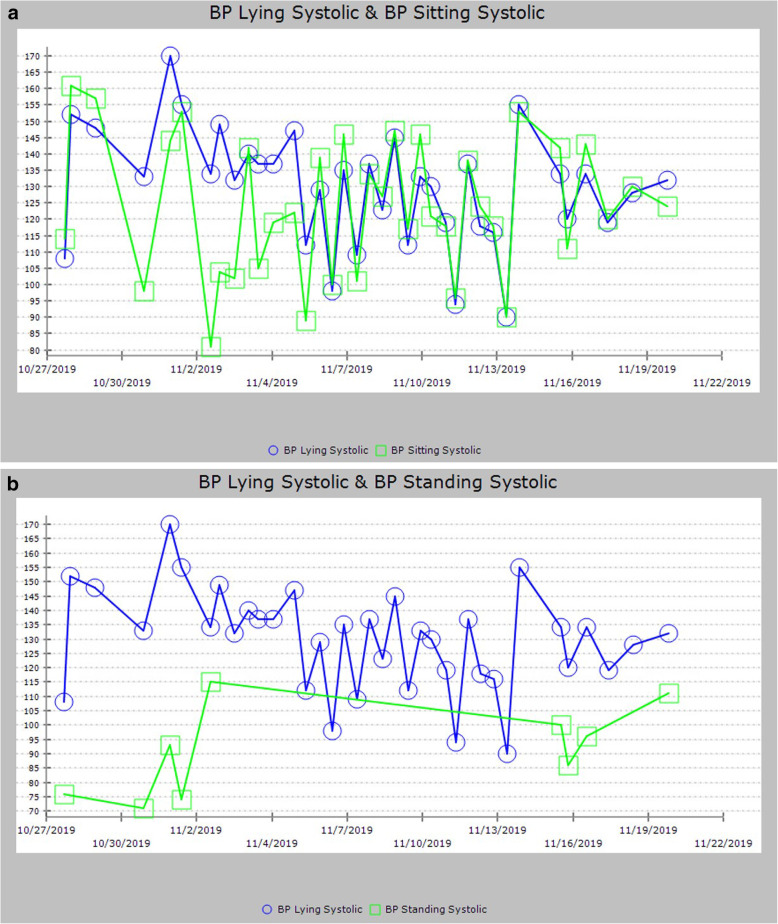
**a** and **b** Measurement of orthostatic blood pressure lying to sitting (**a**) and lying to standing (**b**) over admission. *BP* blood pressure

At discharge, 30 days after initiation of droxidopa, he was still greatly improved in his subjective well-being, stamina in a seated position, and objectively in terms of his baseline SBP, orthostatic measurements (Fig. 5a and b) (no orthostatics were recorded before 27 October 2019 due to profound pre-syncope with attempts), and HR. Impressively, whereas he had seven episodes of bradycardia below 50 during the first 8 days that were recorded on telemetry, after droxidopa was increased to 300 mg three times a day on day 9 there were no more recorded episodes of bradycardia recorded for the following 12 days until telemetry was discontinued. Overall, however, his prognosis at the time of discharge was very poor, and he elected to go home instead of rehabilitation. He did not attend any of his scheduled post-discharge appointments for chemotherapy or oncology. After loss to follow-up, we learned that he died 10 weeks after discharge.

## Discussion and conclusions

This case demonstrates the importance of recognizing the signs and symptoms of amyloidosis, which was our patient’s primary etiology for nOH. We show that in severe, refractory nOH, droxidopa is an option that can be used in addition to midodrine. Our patient had a rapid resolution of both hypotensive and bradycardic episodes after droxidopa was started. We only found one other publication that used both droxidopa and midodrine, and no publications were found that associated droxidopa with a decrease in episodes of symptomatic bradycardia. We feel these are important findings because of the potential for similar patients to improve with the proposed treatment.

Amyloidosis is a rare but important diagnosis to consider when patients manifest classic symptoms such as nOH, peripheral neuropathy, macroglossia, diarrhea, and heart failure. Our patient had a striking presentation for which the diagnosis became especially apparent after an echocardiogram showed a bright, speckled appearance of myocardium. The diagnosis provided relief to our patient and his family after over a year of progressive symptoms that were previously undiagnosed.

The additional important point of our case was that droxidopa can be used as an additional therapeutic for severe refractory orthostatic hypotension as well as reflex bradycardia in AL amyloidosis. Our patient tolerated droxidopa well, and it is shown to have only minimal side effects such as headache, dizziness, nausea, hypertension, and fatigue [8]. In addition to objective improvements in orthostatic hypotension and bradycardia, our patient felt better subjectively. This may be because of the central effects of norepinephrine, where it is important for memory, vigilance, and mood [12].

Droxidopa has been shown to have little effect on resting HR [13], but whether it can prevent episodes of reflex bradycardia has not been investigated. Impressively, our patient ceased to experience episodes of reflex bradycardia when taking the medication. This use of the medication is not documented in the literature; however, we believe it is mechanistically plausible because norepinephrine has a positive chronotropic effect [6]. Therefore, when patients with nOH who have low systemic levels of norepinephrine take droxidopa and replete their norepinephrine levels they may better counteract the vagal response causing reflex bradycardia.

In addition, we used droxidopa concomitantly with midodrine. We only found one other instance of this in the literature [14]. This study showed droxidopa was an effective additional therapy after patients had inadequate response to midodrine monotherapy, and patients reported no side effects to the combination.

In studies of the efficacy of droxidopa, the medication was shown to be effective for only a period of 1–2 weeks [8]. However, our patient experienced a persistent response to droxidopa in terms of preservation of his SBP and quality of life for the remaining 30 days of his hospitalization. We hypothesize that this more prolonged improvement could be due to a synergistic effect between midodrine and droxidopa, which work through different mechanisms. The dose could have been further increased to the maximum of 600 mg three times a day if needed, however, this was not necessary in our case.

One of the major strengths of our case report is the striking response that our patient had to initiation of droxidopa. He had five medical emergencies called on the hospital overhead speaker system for hypotension and bradycardia prior to initiation of droxidopa, but he had none after. He had both subjective and objective improvement in symptoms with droxidopa, and there are many supportive datapoints before and after therapy for SBP and pulse to show this effect (Figs. 3 and 4). Another strength is that we are generating the hypothesis that droxidopa may have a counteractive effect on reflex bradycardia, which could be explored in the future.

Although we are reporting the findings in a case report with one patient in a non-experimental fashion, many of the findings we note suggest a strong possibility of causation that droxidopa was able to correct this patient’s severe, refractory nOH due to amyloidosis. The Bradford Hill criteria is a set of conditions that if met can suggest causation [15]. The first condition met is the strength of the effect. Our patient displayed a strong response to initiation to droxidopa, whereby he stopped suffering from his frequent and severe episodes of hypotension and bradycardia. This effect was consistent over time, during the next 30 days of vital sign measurements. We feel the effect was specific to the initiation of droxidopa because it was the only change that occurred at the time of improvement. There is a strong temporal relationship between the drug and improvement. Further, there is a plausible explanation for the effect. Droxidopa is a prodrug that is converted systemically into norepinephrine. People with nOH who are systemically depleted of norepinephrine will benefit from this increase by having increased vascular tone and will also be able to counterbalance vagal output to maintain a stable HR.

Of course, it is important to consider alternative explanations when considering causal relationships [16]. We considered that our patient may have been volume depleted, although serum creatinine levels improved with diuresis and there was no contraction alkalosis noted on laboratory results. Further, it took 5 days after the cessation of diuretics and giving fluids for these episodes to stop. Bradycardia would not be expected in the setting of volume depletion, and we believe these events to be due to dysfunction of the autonomic nervous system in the setting of high vagal tone that could not be compensated for.

Adrenal insufficiency is another potential confounder, but our patient had normal cortisol levels on two separate occasions and a normal ACTH level. He did receive a dose of dexamethasone on arrival to our ED for his profound hypotension, but he did not improve until just after droxidopa was given, 8 days later. Also, he did not have electrolyte disturbances that would be indicative of this, such as hyponatremia or hyperkalemia. He did receive dexamethasone again later in his hospitalization as part of a chemotherapy regimen, starting day 19, but this is long after he had already improved.

Finally, we also considered his malnutrition as a possible confounding condition that when treated may have resolved his symptoms. He was admitted with a normal BMI, but he had been having weight loss and generalized weakness. We formally consulted our nutritionist of day 1 and started him on PPN. It is possible that on day 8 his nutrition status improved to the point that he no longer had hypotensive and bradycardic episodes, but it is unlikely that there would be such a drastic improvement. Further, the episodic nature of these episodes which always coincided with high vagal tone would be an unusual presentation of malnutrition.

Overall, our format of a case report cannot prove that droxidopa caused the improvement in episodes of severe hypotension and bradycardia, but we think that this is a claim worth further exploration.

Weaknesses are that we only show this effect in one patient, the effect cannot be generalized, and there was no blinding performed. Without blinding and the lack of a controlled experimental setting whereby one variable is changed at a time, it is more difficult to be certain that droxidopa was the sole variable that caused this patient’s improvement in bradycardic episodes. Also, as noted above, our patient was lost to follow-up, so we only have data for 1 month after starting droxidopa.

In summary, our case supports the use of droxidopa in addition to midodrine for severe, refractory cases of nOH due to amyloidosis who have failed midodrine monotherapy. Droxidopa also seems to have prevented reflex bradycardia during episodes of high vagal tone in our patient, which is an interesting area for future exploration. The usage of droxidopa with midodrine was safe and effective both subjectively (well-being and quality of life) and objectively (improved BP, orthostatics, and prevention of bradycardic episodes).

## Data Availability

All data generated or analyzed during this study are included in this published article.

## Abbreviations

ACTH: Adrenocorticotropic hormone
ADL: Activities of daily living
AL: Amyloid light-chain
ASPEN: American Society for Parenteral and Enteral Nutrition
BMI: Body mass index
BNP: Brain natriuretic peptide
BP: Blood pressure
CT: Computed tomography
ED: Emergency department
EKG: Electrocardiogram
FDA: Food & Drug Administration
HR: Heart rate
JVD: Jugular venous distention
nOH: Neurogenic orthostatic hypotension
PPN: Peripheral parenteral nutrition
SBP: Systolic blood pressure
UA: Urine analysis
WBC: White blood cell
